# Sinus Lift and Transantral Approach to Root Fragment Removal

**DOI:** 10.1155/2013/612108

**Published:** 2013-12-25

**Authors:** Andrea Enrico Borgonovo, Federica Rizza, Adele Dudaite, Rachele Censi, Dino Re

**Affiliations:** ^1^School of Oral Surgery, University of Milan, Via Festa del Perdono 7, 20122 Milano, Italy; ^2^Department of Oral Rehabilitation, Istituto Stomatologico Italiano, University of Milan, Via Pace 21, 20122 Milano, Italy; ^3^Department of Periodontology and Implantology III, Istituto Stomatologico Italiano, Via Pace 21, 20122 Milano, Italy

## Abstract

The aim of this case report is to present a case of root fragment removal during planned sinus lift procedure. After failed molar tooth extraction, we chose to retrieve the residual root apex with transantral approach not to damage excessively bone volume. Without changing primary implant rehabilitation purpose, the fragment removal procedure was performed prior to implant placement during necessary sinus lift surgery. Higher visibility of surgical field was achieved. The root fragment residual was removed without an additional surgery appointment avoiding postoperative discomfort. The goal is to underline the importance of being able to change planning during intrasurgical complications. It is most appropriate to operate with safe and simple procedures to reduce surgical discomfort for the patient.

## 1. Introduction

Various treatment modalities are available for replacing a single missing tooth: removable partial denture, fixed partial denture, or dental implant. The choice is influenced by clinical-, dentist- and patient-immanent factors [1], but both dentist and patient increasingly choose to avoid to damage neighbouring teeth and to rehabilitate with endosseous implant [2].

Traditionally, before placing dental implants, the compromised teeth are removed and the extraction sockets are left to heal for 3-4 months [3].

Sometimes complications may happen and it is important to be able to plan again a surgical procedure limiting damage and discomfort for the patient.

The most frequent extraction complication is certainly the root/apex fracture in the alveolus [4]. When it occurs in upper jaw, it is important to manage it to preserve the socket and avoid an oroantral fistula.

Removal of a root through the alveolar opening without the removal of bone from the extraction socket is not always possible and excessive bone removal may result in chronic complications and lack of bone volume for the following implant restoration [5].

In the literature there are some studies about removal of a foreign body from the maxillary sinus [6–9]. These studies suggest to have a clear view of the surgical field and so to use Caldwell-Luc technique that permits the elimination of blind procedures, however, removing a large portion of the anterior maxillary wall. Otherwise, this technique remains invasive and traumatic, with consequent complication rates associated [10], like swelling, infection, orbital hematoma, visual disturbances, infraorbital nerve damage, and oroantral fistula.

In this clinical report, we are presenting a case in which the fragment was not dislocated in the maxillary sinus, but it was retained in the palatal alveolar bone under Schneiderian membrane. The surgeon decided to leave the fragment knowing that it will be removed during the planned sinus lift procedure for endosseous implant rehabilitation.

This decision favourably resulted in a minimal damage for the patient and a reduction of surgical appointment.

## 2. Case Report

**Figure 1:**
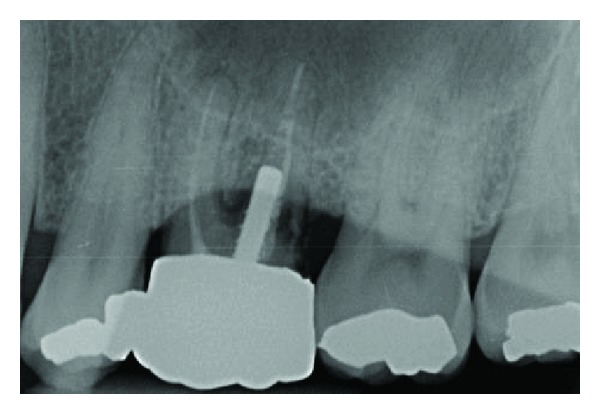
Initial X-ray examination.

A 51-year-old male patient (R.R.), with good general health (ASA 1), nonsmoker, was referred to our department for rehabilitation of the first molar (Figure 1). The tooth was periodontally compromised, with mobility of II degree and furcation interest. It was decided to extract it and in the second phase, after 3-month-healing period, make a single-tooth implant restoration with the bone volume augmentation.

**Figure 2:**
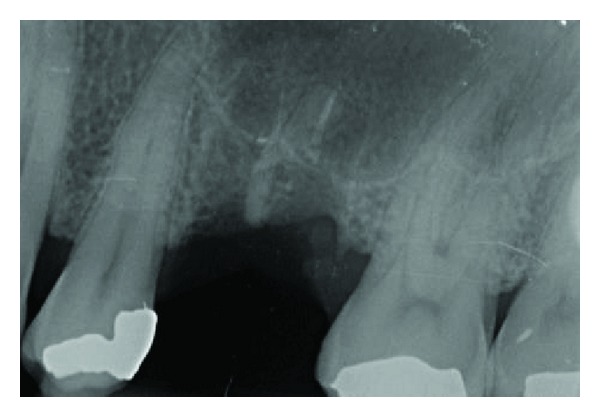
X-ray examination after first extraction: residual root and insufficient bony support.

The first attempt to extract this tooth was made by nonexpert oral surgeon and the palatal residual root was left inside (Figure 2).

Also during a second session with a specialist, it was difficult and complicated to remove the entire root, which was fragile and fell apart.

**Figure 3:**
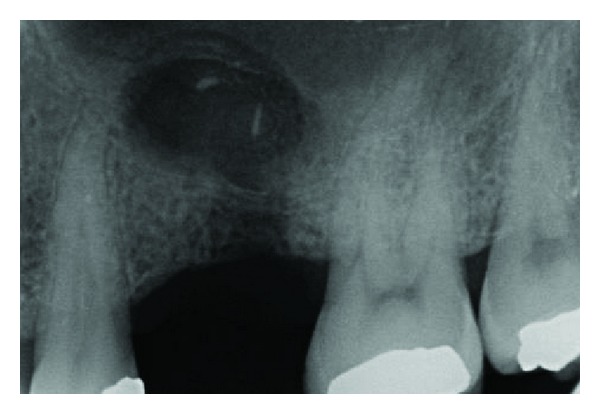
Bone lesion occurred trying to retrieve the residual fragment before sinus lift approach.

To avoid a higher bone loss and oroantral communication (Figure 3), it was decided to leave the last apical fragment in the alveolus. The primary purpose had not changed, and it was chosen to retrieve the residual root apex during sinus lift procedure, because antrostomy approach would help to remove it with a major visibility of the surgical field.

**Figure 4:**
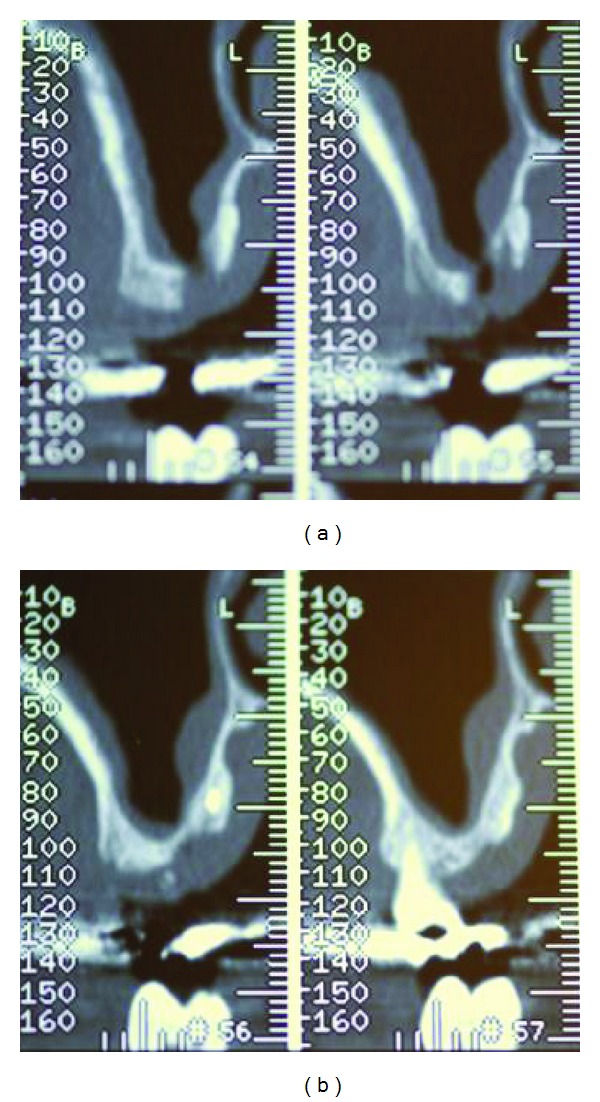
((a), (b)) Radiographic exams (paraxial images) also revealed not only the residual root apex in the palatal side, but also insufficient bone volume.

After the healing period, a further accurate radiographic exam was necessary. As for any other sinus floor elevation case, reformatted computer tomography (CT) scans were required to examine insufficient bony support (Figure 4).

**Figure 5:**
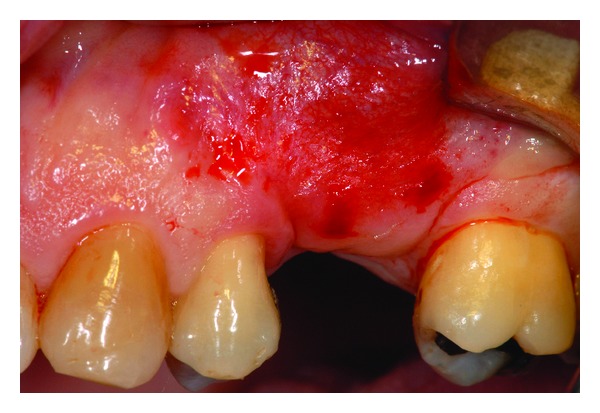
Surgical site.

**Figure 6:**
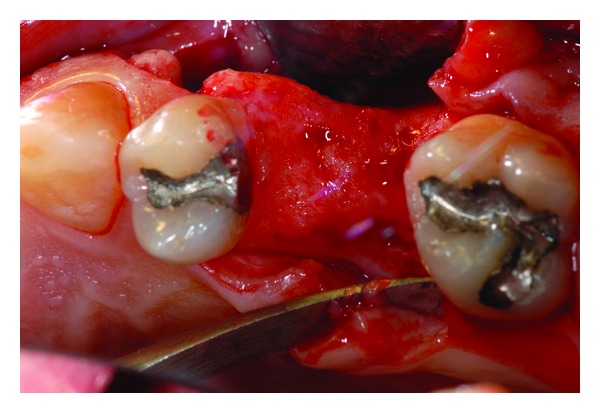
Alveolar bone exposed.

This exam also revealed the palatal position of the residual root apex. Surgery was performed under local block anesthesia (posterior superior alveolar nerve, greater palatine nerve, and buccal infiltration from the canine to the first molar). An intrasulcular and crestal incision was performed and a full-thickness mucoperiostal flap was reflected to expose the maxillary and palatal wall (Figures 5 and 6).

**Figure 7:**
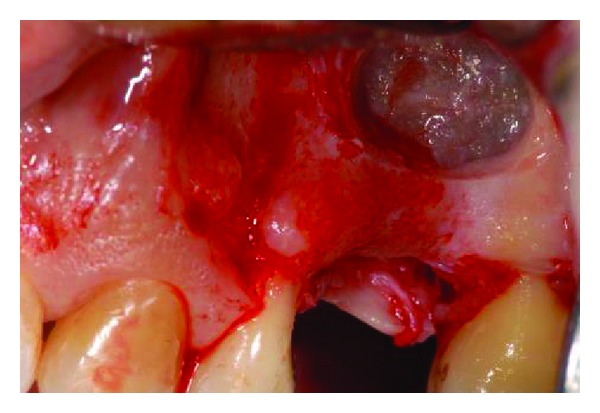
Antrostomy on the lateral maxillary wall.

An antrostomy with 5 mm diameter was made approximately from the distal root of first premolar to mesial root of second molar (Figure 7).

**Figure 8:**
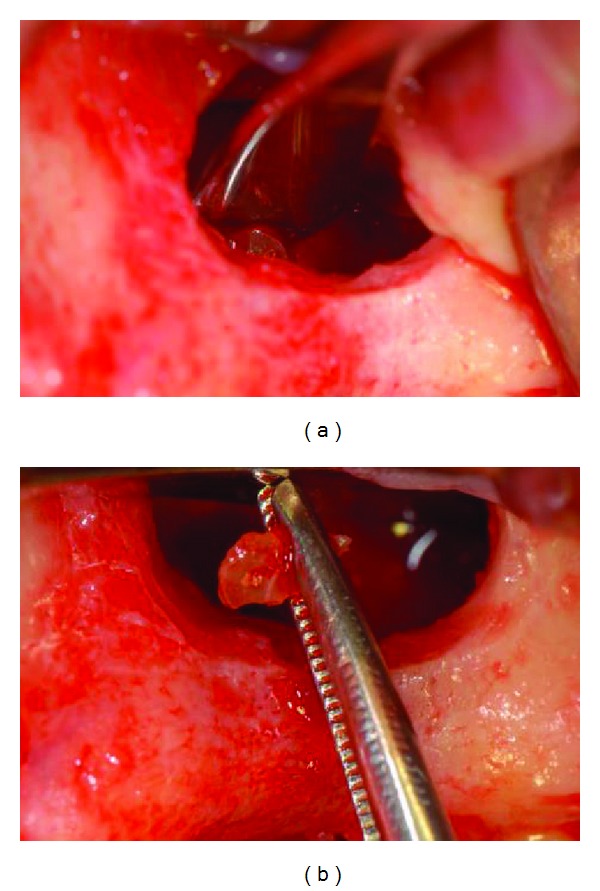
(a), (b): Intra-operative images during retrieval of residual root apex through the antrostomy.

The membrane was elevated from the bone and we could see the root fragment adjacent to the sinus floor. The residual was removed without difficulty with hemostatic clamps (Figures 8(a) and 8(b)).

**Figure 9:**
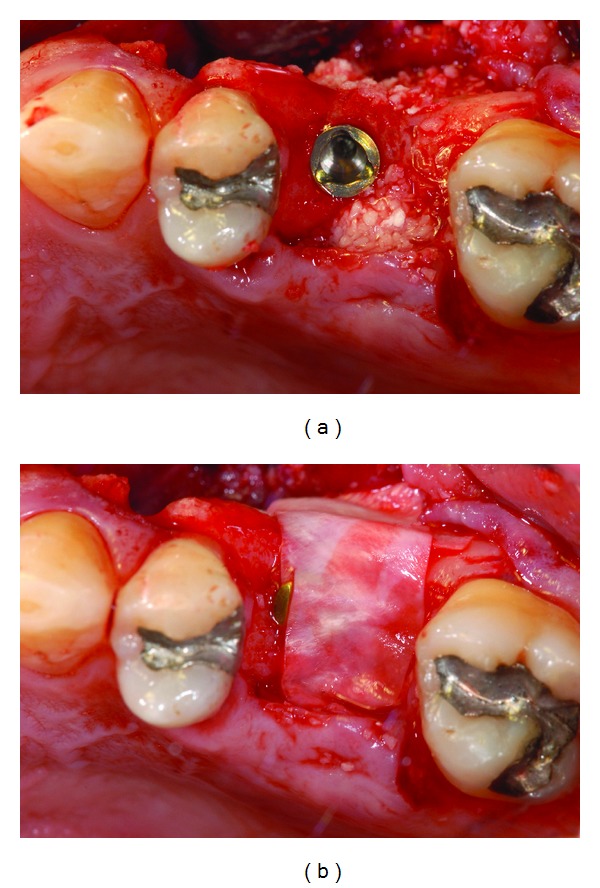
(a) Insertion of graft material; (b) collagen membrane positioned.

Afterwards, implant was placed using a conventional approach: drills were used to prepare the fixture bed and the implant of 4,3 Ø in diameter and 11,5 mm of length (NobelReplace Select, NobelBiocare, Göteborg, Sweden) was installed protecting elevated sinus membrane. The insertion torque was of 35 N/cm, measured with a manual torque wrench by the operator. Particulate graft material (Geistlich Bio-Oss) was inserted between Schneiderian membrane and sinus floor and also between implant and vestibular tissue to fill the gap (Figure 9(a)).

In the lateral bony windows collagen membrane was installed to reinforce and keep stable graft material (Figure 9(b)).

The wound was closed primarily and detached sutures were positioned.

Pharmacological management was with antibiotic therapy (oral amoxicillin and clavulanic acid 875 mg + 125 mg every 8 hours) and nonsteroidal anti-inflammatory drugs (ibuprofen 600 mg, as needed by the patient).

Sutures were removed after fifteen days and no postoperative complications were present. No signs of infection, inflammation, or maxillary sinusitis were detected.

**Figure 10:**
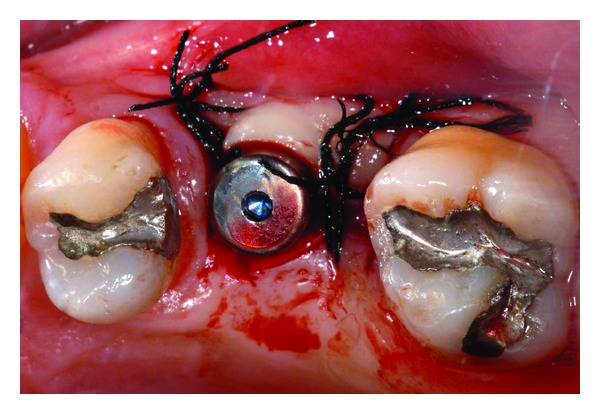
Reopening after 3 months.

**Figure 11:**
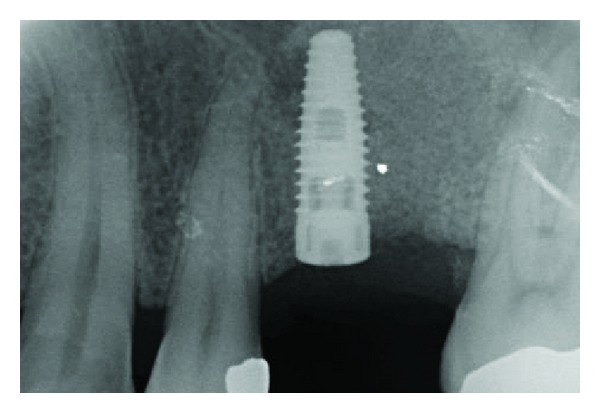
X-ray control after 3 months.

**Figure 12:**
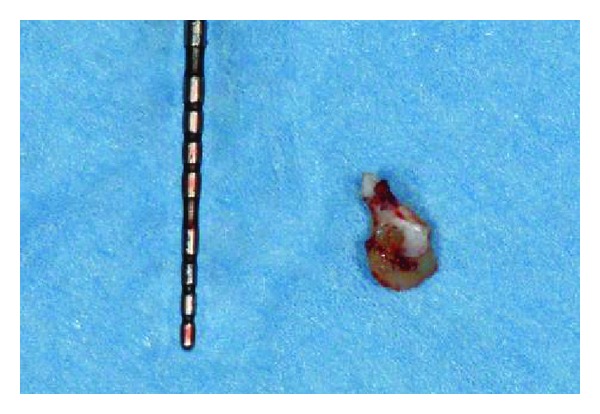
Retrieved residual root fragment.

After three-months, the second-stage operation was carried out to expose the fixture and cover screw was placed (Figure 10). Radiograph examination showed (Figure 11) correct osteointegration, displaying good bone filling and the successful fractured apex retrieval (Figure 12).

## 3. Discussion

Extraction is the most common surgery performed in the dental office. Although most cases are simple, complications can occur.

The fracture of a root is the most frequent complication of exodontia [11]. It occurs accidentally and may cause severe problems as infection, residual cyst, and, in case of displacement of the fragment, an oroantral fistula [12].

In endodontically treated teeth, fracture resistance decreases, and the amount of stress in dental tissues increases in the face of functional forces. These stresses are vertical and oblique forces, which are the basis of masticatory function and increase the possibility of fracture both of crown and root. The restoration types play an important role in the clinical prognosis of these endodontically treated teeth [13]. Many studies have revealed the ideal restorative approaches that increase fracture resistance of remaining dental tissues after endodontic treatment and enables minimal stress transmission [14, 15]. However, when there is a strong patient's interest on preserving a compromised tooth, it must be clearly underlined that endodontically treated teeth are more weak and with a high probability of fracture and possible complications.

In case of the fractured fragment retrieval the visibility of surgical area takes a very important role.

Trying to access the apex with a blind procedure could determinate unjustified aggressive treatment with worse prognosis of alveolus healing influencing bone insufficiency in the area.

Furthermore, the inadequate bone volume of our patient required a surgery to enhance it for implant placement and a higher bone volume loss had to be prevented by all means. It was decided to perform an antrostomy procedure with implant rehabilitation and simultaneous retrieval of the root fragment. The necessity of a sinus lift surgery determines adequate healing time (in our case it was 3 months) to be sure to achieve the correct primary coronal stability.

There are cases present in the literature of foreign bodies displacement into maxillary sinus, because of the increased oral implant migration. If the search is restricted just to fractured root, the results highlight correlation with extraction of maxillary third molars.

Even if a foreign body is a root or an oral implant, the migration of foreign bodies into paranasal sinuses is certainly a severe complication and it needs a proper procedure for its retrieval to avoid chronic infections. Most authors chose Caldwell-Luc technique [16–18]. This approach provides direct access to the sinus but may result in the partial loss of functions of the sinus. For that reason the most frequently used technique is the functional endoscopic sinus surgery (FESS) [19]. This technique is minimally invasive, recreating competent sinus ostial patency, and provides access to drain and clean the cavity exudates.

In case of a foreign body migration into sinus, it is important to emphasize that patients do not always show symptoms. In the literature there are reports of asymptomatic cases [16] and reports of patients with infections, such as acute and chronic sinusitis [16, 20, 21]. But it would be appropriate to remove all foreign bodies present in the maxillary sinus, because of the interference with the sinus clearance and the inflammatory response of the sinus membrane.

In our case the fractured root was not in the sinus but still retained by the sinus membrane.

Lifting up the Schneiderian membrane permits to open the surgical area, with a better view and to reach the residual fragment with noninvasive instrument like haemostatic clamps.

With this work, we want to underline the importance of being able to change planning during intrasurgical complications. It is most appropriate to operate with safe and simple procedures to reduce surgical discomfort for the patient.
